# The MultiSOCIAL Toolbox: An open-source toolkit for advancing multimodal interaction research

**DOI:** 10.3758/s13428-026-03176-w

**Published:** 2026-09-18

**Authors:** Veronica Romero, Tahiya Chowdhury, Alexandra Paxton, Muneeb Nafees

**Affiliations:** 1 Psychology Department, Colby College, 5566 Mayflower Hill Dr., Waterville, ME 04901 USA; 2 Davis Institute for Artificial Intelligence, Colby College, 4614 Mayflower Hill Dr., Waterville, ME 04901 USA; 3 Computer Science Department, Colby College, Waterville, ME USA; 4 Department of Psychological Sciences, University of Connecticut, Storrs, CT USA; 5 Center for the Ecological Study of Perception and Action, University of Connecticut, Storrs, CT USA

**Keywords:** Multimodal analysis, Communication, Video analysis, Audio analysis, Transcription, Open-source methods, Friendship

## Abstract

Human interaction is intrinsically multimodal, in that it unfolds across different kinds of behaviors (or *modalities*). However, many researchers have found it difficult to systematically investigate the richness of multimodal communication due to its theoretical and technical complexity. To help overcome some of the technical hurdles, we introduce an open-source single-platform solution: the MultiSOCIAL (*Multi*modal time*S*eries *O*pen-Sour*C*e *I*nteraction *A*nalysis *L*ibrary) Toolbox. The Toolbox enables any researcher who has video files to extract time-series data in three modalities: body movement to quantify non-verbal behavior through a pose estimation algorithm; transcripts of what was said during an interaction through the use of an automatic speech recognition system; and acoustic prosodic characteristics of speech through the use of audio signal processing. The toolkit uses existing gold-standard open-source tools, and we have brought them together in a freely available graphical user interface (GUI) that requires no coding and runs exclusively on the user’s machine. To provide a concrete use-case, we present an analysis of a new dataset collected by undergraduate students as part of a seminar class. Here, we specifically analyze the movement data from the study to exemplify the complexity available in video data from just *one* modality. We find that friends show higher interpersonal bodily coordination than strangers and that this coordination is both more stable and more complex throughout a simple affiliative conversation. We end with some practical recommendations for using the Toolbox, including how to best set up experiments to get the most accurate data possible to maximize data quality.

Human interaction is fundamentally *multimodal* – that is, it incorporates aspects of communication (or *modalities*) such as posture, emotion, language, and non-linguistic speech. Each person has distinct abilities, histories, and cultures that shape their unique communication dynamics, but as a species, we have communication systems that span visual, auditory, and even tactile domains. During naturalistic conversation, communication modalities are stitches of a single tapestry: You *can* isolate them, but in doing so, you lose the richness of the whole.

Theoretical and practical constraints have traditionally limited the quantitative study of communication to just one modality at a time. On the practical side, recording and extracting multimodal time series has historically been limited to labs with expensive equipment and/or extensive human effort. On the theoretical side, dominant linear approaches to psychology have suggested breaking apart the whole of communication into more tractable parts, under the assumption of linear decomposability.

Fortunately, recent decades have seen movement on both fronts. Ongoing advances in computing power and storage capacity – especially over the past decade or so – have reshaped the practical side of the equation, empowering researchers to extract high-quality communication time series from commercial-quality audiovisual equipment. This has been accompanied by rising interest in dynamical theoretical approaches, especially in so-called *4E cognition* (embedded, embodied, extended, and enactive or ecological cognition) that situates cognition as emerging from the brain-body-environment system (for more on 4E cognition, see Newen, Gallagher, & De Bruin, 2018).

Our goal in this article is to introduce the *Multi*modal time*S*eries *O*pen-Sour*C*e *I*nteraction *A*nalysis *L*ibrary (*MultiSOCIAL*) Toolbox, an open-source suite of tools designed to invite even more scholars into this area. We are multidisciplinary scholars actively engaged in the study of human interaction, and we created the Toolbox to share the kinds of force-multiplying tools that have so far been limited to those with programming expertise. In doing so, we hope to provide a shared toolkit for multidisciplinary scholars focusing on human interaction and thereby accelerate cross-disciplinary advances.

## Data extraction methods for interaction research

Research on human interaction has a rich history in behavioral and social sciences. Here, we briefly review some of the methods that have been used to date in order to situate the MultiSOCIAL Toolbox within this landscape.

### Hand-coding

One of the earliest methods for interaction research is *hand-coding* – that is, human annotations of interactions. Hand-coding is often highly labor-intensive, requiring annotators to spend up to ten minutes to annotate a single minute of data (Kreuz & Riordan, 2018). Because it is human-driven, however, hand-coding is quite flexible: Any facet of interaction can be coded, as long as the research team can devise a codebook and training that leads to sufficient reliability.

Some forms of hand-coding can be quite narrow, like marking specific changes of body movement or sounds from one video frame to another (Condon, 1970). Some coding can be simple counts of a target behavior during a given segment of time (Murphy, 2005). Other systems are more broad and incorporate multimodal phenomena. For example, the Specific Affect Coding System (SPAFF) is a holistic rating system that uses all available communication patterns (including tone, facial expressions, and linguistic content) to categorize emotion during interaction (Coan & Gottman, 2007).

All forms of hand-coding have a trade-off between person-hours and complexity. Methods that rely on tallying the appearances of a single clearly specified behavior will take substantially less time than those that rely on multiple facets of behavior or carefully annotating multiple components. This creates a real problem for those with limited resources, but even those with sufficient personnel may look for alternative methods to speed up their research pipeline.

Moreover, hand-coding can be constrained by human bias and inconsistency. For example, even in a coding schemes with very clear training methods, prior research has demonstrated variance across coders based on demographic group membership and has called for improving diversity across coders (Babcock & Banks, 2019). While no research methods are truly value-neutral, it is critical to keep in mind that some hand-coding schemes may be more subject to bias and inconsistency than others.

### Automated methods

Technical advances have provided new options for quantifying interaction. For example, automated motion analyses have grown from requiring highly specialized motion-capture systems to infrared video game cameras to standard commercial video cameras (Romero et al., 2017). Automated language analysis has evolved over time, too, from percentages of specific strings (Tausczik & Pennebaker, 2010) to methods based in large language models (Zhou et al., 2024).

However, automated methods face their own limitations. First, although they often require a lower investment of person-hours compared with hand-coding, automated methods have historically required other sorts of investments – usually in specialized training (e.g., programming), expensive equipment (e.g., motion-trackers), or both. Second, they are often limited to a single communication modality (e.g., movement, language), due in large part to the different recording tools and data required by different kinds of analyses. In other words, most specialized data-collection methods have been designed to record only one modality at a time, and most data-intensive analysis methods to date have been limited to being able to analyze only one or two time series at a time.

This separation of modalities has resulted in fairly siloed literatures within areas that tend to use automated quantification methods. Most studies – even on multimodal corpora – will focus only on one modality in any given research paper. However, meaning is notoriously multimodal and context-specific (e.g., Attardo, Eisterhold, Hay, & Poggi, 2003; Schifanella, De Juan, Tetreault, & Cao, 2016). We can only come to more fully understand the meaning of an interaction by considering these channels in relation to one another and the communication context at large. We hope the Toolbox will lower the cost of entry to multimodal interaction research and eventually make it easier for researchers to quantitatively disambiguate whether, say, a specific smile is amusement, sarcasm, or sympathy.

## An open-source Toolbox for quantifying interaction

The MultiSOCIAL Toolbox brings together existing tools for analyzing multimodal data (e.g., video recordings capturing social communication) in a single GUI (i.e., *graphical user interface*) to facilitate programming-free data extraction. Currently, the Toolbox supports video, audio, and transcription analyses. We consider these analysis processes as they are typically used for understanding behaviors during social communication using audio (i.e., acoustic-prosodic related speech features) and video (e.g., posture, movement), along with audio-based language extraction (i.e., transcripts). We recognize, of course, that not everyone will be interested in multimodal processes and that not all studies have been designed to investigate multimodal dynamics. The Toolbox has a modular design to extract as many or few of the provided modalities as desired.

### Design considerations

Our research team has a variety of different experiences with technical and computational research pipelines, but we are unified in our interest in understanding social interaction. Given this, we wanted to combine our knowledge of how people already work with these data and some of the qualities that make other tools most helpful. We present some of the overarching design considerations first, before describing the current tools in the Toolbox.

#### Efficient local processing for all

Many computational tools already exist to help researchers extract and analyze data from video and audio, and they enable incredible data-rich analyses. These tools are ideal for researchers who already have specialized knowledge and computational resources, and the MultiSOCIAL Toolbox will not replace those tools. However, for those who are new to the field or who do not have those abilities, there are several bottlenecks that limit wide adoption of existing tools outside of computational research fields.

First, even though these tools are made widely available as open-source software, they often require extensive programming knowledge to set up and to write a routine process for reading, analyzing, and saving extracted information. Second, it can be difficult and costly to obtain the hardware demanded by cutting-edge artificial intelligence-based techniques for these processes, such as extracting human movement from frames in a video or transcribing an audio clip that spans over 2–4 min. High-resolution video data promises better performance for these feature extraction techniques, but such data also requires large amounts of computational power for preserving context. To be able to use such tools effectively, researchers need to invest in appropriate hardware and secured data storage, which opens new issues about research data management, privacy, and equity.

The MultiSOCIAL Toolbox tackles this issue by using memory- and storage-efficient alternatives for these methods while prioritizing accuracy. Importantly, all information from an input file can be extracted while running the processes entirely locally. By “local,” we mean that all of the data and processing stays on the hardware running the MultiSOCIAL Toolbox, typically on a laptop with standard processing power (CPU) and reasonable memory (RAM) capacity. No data are sent to a company or any other third party. This helps protect participant data ethics and (in our experience) helps mitigate concerns about data security raised by our universities’ ethical review boards. It also means that the processes can run without an Internet connection once it is installed, maximizing flexibility and allowing the analyses to be run even on isolated computer systems.

Relying on local processing is a crucial design choice, since many existing tools available for multimodal behavioral data analysis require specialized graphical processing units (i.e., *GPUs*) to process videos and extract behavioral features. In the MultiSOCIAL Toolbox, we include tools optimized to run on standard computing devices through architectural changes and efficient hardware use. Due to this design strategy, the Toolbox allows extracting pose information or transcription using a single local computer with a standard 16 GB of memory.

#### Maximizing ease of use

Many of these tools are available as user-friendly programs for people to use, but they require separate operating environments. Because we wanted MultiSOCIAL Toolbox to facilitate multimodal analyses of verbal and non-verbal modalities, anyone can use as many (or as few) tools in the Toolbox as they might want. It runs entirely in a Python-based environment (Van Rossum et al., 2007), which limits the need for cross-language dependency between different environments. It is customizable and modular by design, which means it can include and support many different tools in the future. (As we describe further in the “Tips and Tricks” section later in the article, we also invite others to help us add tools through community development.)

**Fig. 1:**
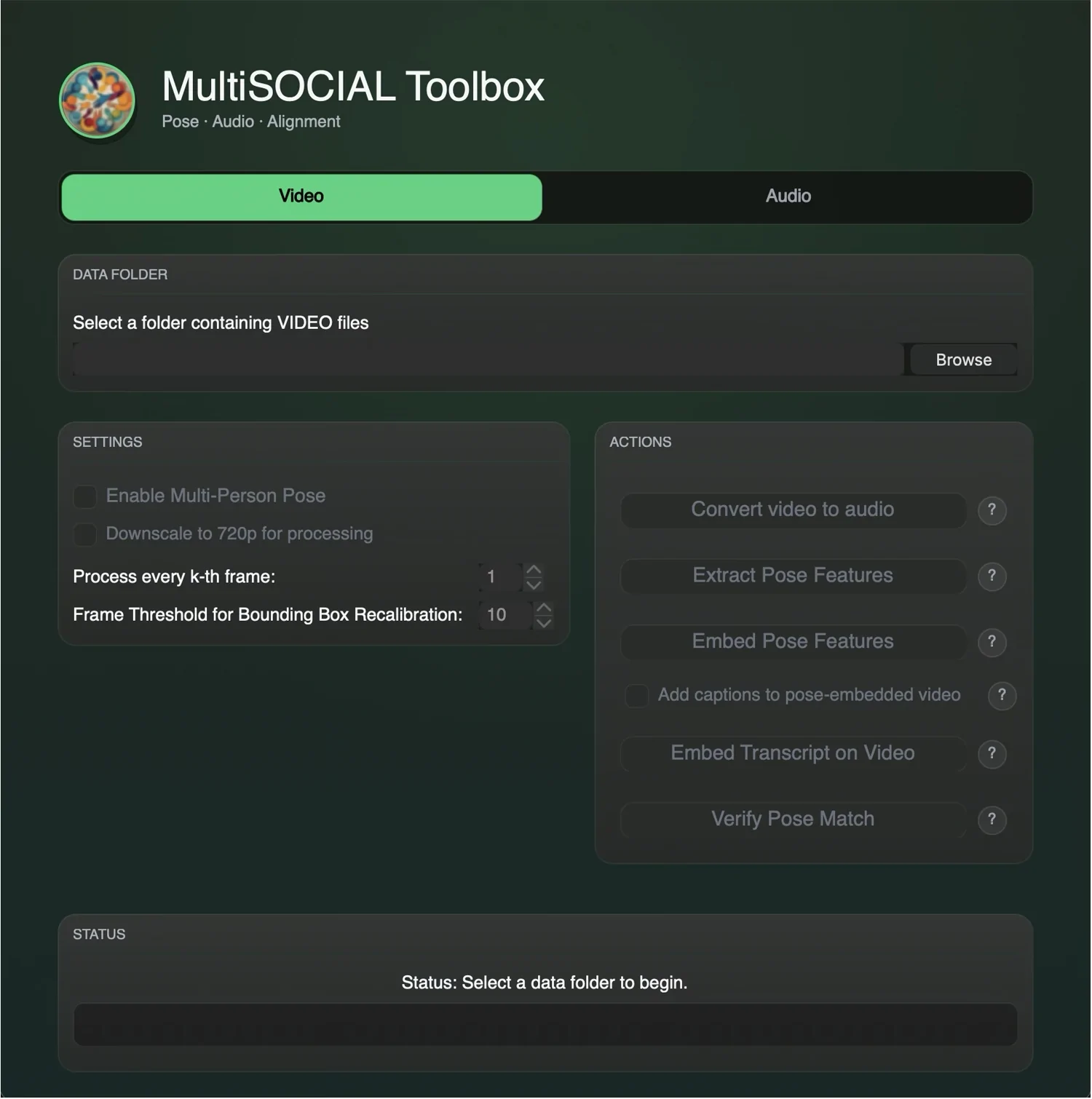
Screenshot of how the application’s GUI appears on macOS

By wrapping all of these tools in a single GUI (see Fig. 1), we hope the Toolbox will be useful to a wide range of scholars and skill levels. All tools can be accessed and utilized through a minimalistic user interface, with no programming required. We hope this lowers barriers to entry and promotes wider adoption of these tools.

#### Seamless integration with common research practices and commercially available hardware

We wanted the MultiSOCIAL Toolbox to work alongside existing practices and tools. In our experience (from our own labs and in discussing with other researchers), most social interaction data are collected as videos, even if any given research team only plans to analyze one modality at a time. While automated tools exist today that can extract information from multiple modalities from video data, our goal was to make the Toolbox both simple to use and consistent with current practices. We approached this by finding ways to help users maximize the use of their video-based input data from commercially available video recording tools.

### Current functionalities

**Fig. 2:**
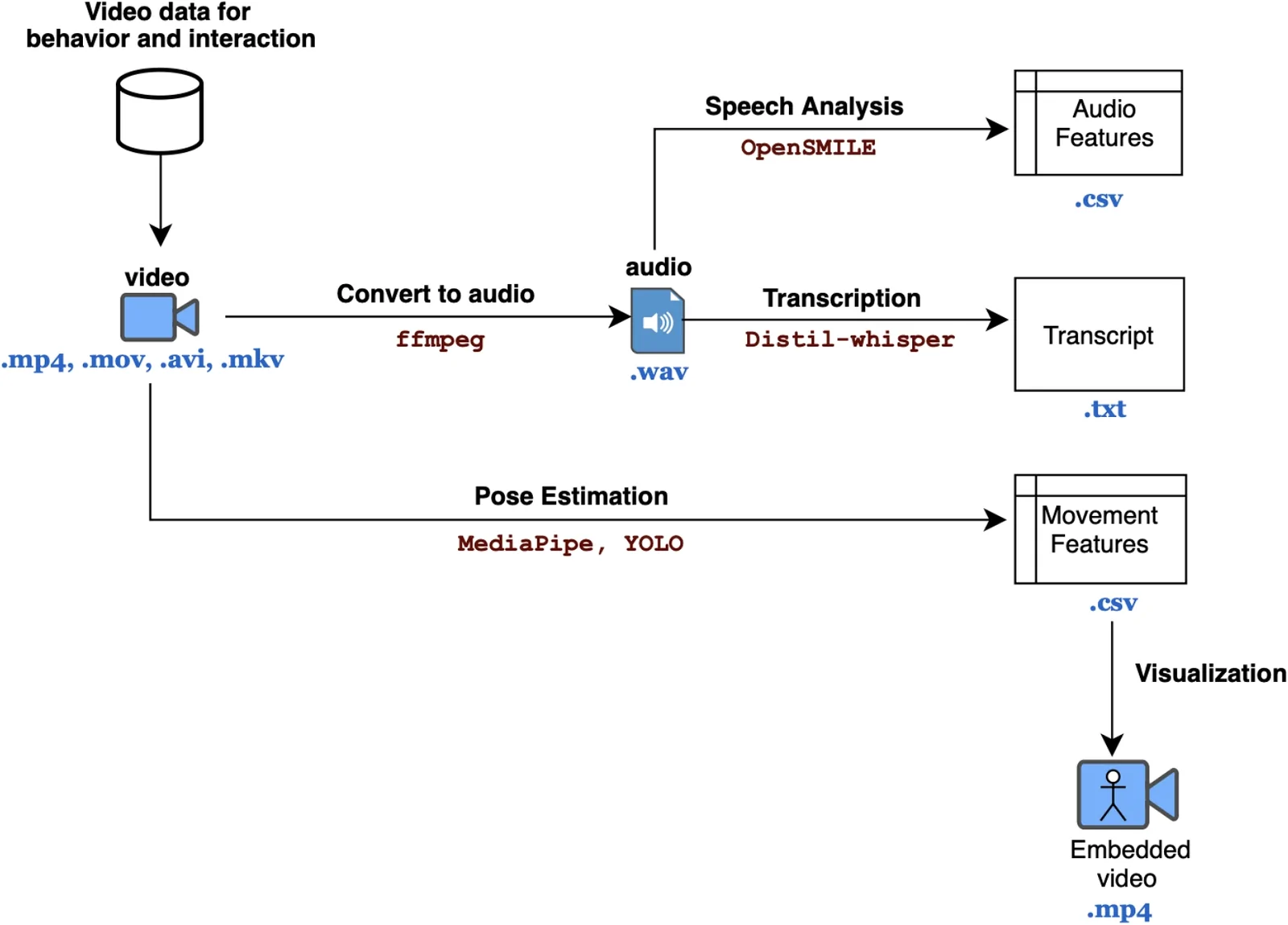
System diagram of MultiSOCIAL Toolbox. Researchers with video or audio data captured for behavioral or interaction research can extract pose, audio, and transcript from an input video file. We include in parentheses all import and export file types, current as of the time of publication; please check current documentation for updates

Below we describe the current functionalities of the Toolbox to support behavioral research and analysis (see Fig. 2).

#### Pose estimation for movement analysis

The MultiSOCIAL Toolbox supports movement analysis by using Mediapipe (Lugaresi et al., 2019). MediaPipe is a well-established framework that integrates BlazePose, a lightweight pose estimation method developed by Google Research (Bazarevsky et al., 2020). While there are other pose estimation models, we chose MediaPipe because it detects  10% more body points and is faster than OpenPose, the dominant alternative model (Chung, Ong, & Leow, 2022). Currently, the Toolbox only provides support for MediaPipe.

From a user-selected video file, the MultiSOCIAL Toolbox will extract the frame-by-frame locations of 33 body landmarks (e.g., right shoulder, left elbow) in *x*,*y*,*z* coordinates. These coordinates – along with a visibility score that can be used as a proxy for the algorithm’s confidence in the estimate of the landmark – are then exported into a CSV file, with one row per frame. Importantly, a row is written whenever a pose is detected in a frame; frames with no detected pose are simply left out rather than filled with a blank row. Because the pose estimation algorithm works on a frame-by-frame basis, users should make sure to know the frame rate (or sampling rate, typically in hertz [Hz]) of their video camera, since that will be the fastest or densest sampling rate available to them for the analyses.

In order to perform this process on a computer that a typical behavioral sciences lab might have, the Toolbox combines a real-time person detector using YOLOv5s, which is an efficient variant of YOLOv5 (Jocher, 2020) ideal for running on edge devices. Then, for each person detected in each frame, the Toolbox uses MediaPipe for keypoint estimation. Typically, commercial video cameras – such as those often used in behavioral research – record at approximately 30 frames per second, yielding a time series of motion for each detected person sampled at 30 Hz.

Using the camera’s standard frame rate can yield very detailed tracking of movement but will take longer to process. Therefore, the Toolbox provides options for reducing processing time by reducing data density. The first option allows users to downsample the sequence of frames to their desired sampling rate using the optional parameter *frame skip*. By default, this is set to “1,”, but can be updated. For example, selecting two means that every second frame will be skipped (retaining frames 1, 3, 5, etc.). The second option lets users reduce video quality. In this option, *x* and *y* dimensions of each frame can be optionally resized to a smaller resolution for spatial downsampling. This design choice involves a trade-off between faster per-frame processing and a small decrease in accuracy (for example, downsampling the video resolution to 720 px). The resulting frame will have a downgraded resolution and this choice can thus affect the downstream task performance.

Next, the Toolbox does a data-quality check for the people it detects. Bounding boxes localized with a low confidence score (i.e., visibility) are discarded using a parameter *confidence threshold*. Locations of the detected people are passed to the MediaPipe pose estimation module. By default, the Toolbox has purposefully been configured with the most lightweight pose estimation tool to reduce computational demand. This step returns 33 landmarks per person in a frame, each with its *x*, *y*, and *z* coordinates and the visibility score (a value between 0.0 and 1.0), yielding a total of 132 columns for each person detected in the video. The resulting data are saved to a CSV file on the user’s local machine.

Because many social-interaction experiments include more than one person in a single video, the MultiSOCIAL Toolbox supports both single- and multi-person videos. To track each person across different frames and ensure their corresponding data is recorded in the correct manner, we developed a custom pipeline to track each individual consistently and assign them an ID across frames so that each resultant CSV file contains data from a single individual for longitudinal analysis.

In addition to the CSV, the Toolbox can provide an additional output for the video analysis: The “Embed Pose” option will generate a video file that overlays the extracted keypoints over the raw video. In our experience, the pose-embedded video serves two purposes. First, it provides a powerful visual for science communication, helping your audience to understand what your analyses are capturing more readily. Second, it provides a helpful tool for data verification, helping the researcher to validate the extracted data with human oversight to identify systematic issues with data extraction and algorithmic validity.

To further support quality control, the Toolbox offers a “Verify Pose Match” option that checks whether the generated pose-embedded videos remain internally aligned with the extracted CSV files. Although the pose overlay is generated from the extracted coordinates, this verification step is useful for detecting practical workflow errors that can occur during batch processing or repeated analysis runs, such as stale output files, mismatches between single- and multi-person outputs, incorrect CSV-to-video pairing, frame-index mismatches introduced by frame skipping, or errors in matching tracked person IDs to the correct CSV files. The tool matches each embedded pose video to the appropriate CSV set based on its output mode: Single-person videos are matched to *_ID_*.csv files, whereas multi-person videos are matched to *_multi_ID_*.csv files. It also reads the extraction stride saved during pose extraction so that skipped frames are handled consistently during verification. The tool then computes a landmark hit rate by checking whether each keypoint coordinate stored in the CSV falls within a small spatial window of the corresponding colored keypoint rendered in the embedded video. The verification output is written to a verification/ folder and includes per-video reports, reference images for frames with the lowest agreement, and a summary file aggregating results across videos. Thus, this option should be understood as an internal consistency and workflow quality-control check, rather than an independent validation of MediaPipe’s pose-estimation accuracy.

#### Audio feature extraction for non-linguistic speech analysis

The MultiSOCIAL Toolbox uses OpenSMILE (Eyben, Wöllmer, & Schuller, 2010) for audio feature extraction. OpenSMILE is an open-source Python-based toolkit for audio processing and analysis.^1^). Of the three available options in OpenSMILE, we use the ComParE 2016 feature set for its appropriateness to behavioral and psycholinguistic analysis. Currently, no other audio extraction feature sets are supported by the Toolbox.

The Toolbox currently accepts standalone audio files. Users can provide these from their own audio recordings, or users can use the Toolbox to pull audio files from integrated audio-video files using the “Extract Audio” function. The Toolbox loads the audio file at its original sampling rate, so knowing the original audio sampling rate of the video or audio will be helpful for understanding the resulting data.

Next, the Toolbox extracts prosodic, spectral, and voice quality measures from the selected audio file. Although OpenSMILE includes thousands of different audio features, our Toolbox strategically includes the 65 audio features that are part of the “low-level feature” portion (e.g., pitch, energy, jitter, shimmer). Values for each feature are provided once for every 10 ms of audio (i.e., 100 Hz). The Toolbox then saves the resulting values to a CSV file with the audio features for each 10-ms sample, in which each row includes one sample and each column includes one feature.

Importantly, as readers may have inferred, the sampling rates of the audio and movement features are not aligned. By nature of the two phenomena, the audio features are sampled much more densely (default: 100 Hz) than the movement features (default: 15 Hz, given built-in downsampling from industry-standard 30-Hz recording). Although researchers interested in multimodal data analysis may already be aware of these different timescales of movement and speech, we want to flag this for those who may be new to integrating these modalities.

#### Automated transcription for language analysis

The MultiSOCIAL Toolbox implements automated speech transcription using Whisper Large V3 Turbo, a fine-tuned version of a pruned Whisper large-v3. The model is much faster because it removes some intermediate layers, but it has minor quality degradation as a result. At present, the Toolbox only uses the version available through Hugging Face (Radford et al., 2023). As with the audio feature extraction, the user can select a standalone audio file or extract audio from a video file.

Due to the computational demand of this model, we allow the Toolbox to use GPU (or comparable Apple Silicon alternative) if available on the local device. If no GPU is available, the Toolbox will fall back to using CPU for processing the audio file. We also make some additional modifications in the speech recognition pipeline (e.g., smaller batch size, shorter chunk length) to process the audio recordings on device while reducing memory demand.

For each wav file, the pipeline produces a single transcript saved as a TXT file. When speaker diarization is enabled, the Toolbox uses PyAnnote (Bredin, 2023; Plaquet & Bredin, 2023) to assign speaker labels, and the transcript is saved in a turn-by-turn format with segment-level timestamps. The main transcript file does not include word-level timestamps by default; when needed, word-level timing can be obtained and temporally aligned in a post-processing step with audio and movement features and their file-level timestamps.

For each audio file, the Toolbox produces a readable transcript saved as a TXT file and a corresponding SRT caption file. When speaker diarization is enabled, the Toolbox uses PyAnnote (Bredin, 2023; Plaquet & Bredin, 2023) to assign speaker labels; the TXT and SRT outputs are saved in chronological speaker-turn segments with segment-level timestamps. Word-level timestamps are optional and are saved separately in a _words.json file when requested or when feature alignment is needed.

The Toolbox’s *Align Features* step combines optional word-level transcript timestamps with timestamped OpenSMILE features. For each word, it aggregates the acoustic-feature frames associated with that word’s time interval and saves the resulting word-level dataset as an _aligned.csv file. Pose features are exported separately with processed-frame indices and the frame-sampling setting used during extraction. Researchers can therefore map pose observations to the recording timeline and integrate movement, acoustic, and transcript data in downstream analyses. Direct audio–movement alignment is not currently automated within the Toolbox.

## An example study for applying the Toolbox

In this section, we demonstrate the Toolbox in addressing a topic of interest to us – interpersonal movement coordination. Because the Toolbox is designed to support quantification, it can easily accommodate a wide range of specific questions and theoretical perspectives, but this study provides an opportunity for readers to better understand the possibilities available through using one modality of the Toolbox. Our example comes from a study conducted by undergraduate students as part of an undergraduate seminar class at a liberal arts school in the US. In this example, we specifically analyze the movement data from the study, given the particular complexities of grappling with multivariate data.

Although the Toolbox is designed with multimodality in mind, the scope of the current work does not lend itself to full multivariate analysis of data. This is especially the case given that quantitative multimodal research methods are still emerging, with very few studies to date truly integrating multiple modalities; as mentioned earlier, most research so far has tended to study multiple separate modalities from the same dataset. Scholars in this domain are still actively searching for paradigms that will promote human-interpretable and theory-driven multimodal research. Ultimately, we hope the Toolbox will help contribute to this search by providing a straightforward and unified tool for data extraction. In the meantime, we wanted to highlight how it could be a helpful tool for pose estimation data extraction even for those without extensive technical experience.

When people talk to one another about neutral or positive topics, they often become more similar in their movement patterns over time. Researchers refer to this phenomenon by many names, including interpersonal movement synchrony or interpersonal movement coordination (e.g., Cornejo, Cuadros, Morales, & Paredes, 2017). A dominant view in this work finds a positive and bidirectional link between movement similarity and rapport; this holds both for very stereotyped movements (e.g., finger tapping between participants and experimenters; Hove & Risen, 2009) and self-directed natural movement (e.g., gross movement of clients and therapists during psychotherapy sessions; Ramseyer & Tschacher, 2011).

Increasingly, however, researchers have been questioning whether interpersonal movement coordination always emerges in the same way across all interactions. For example, prior work has shown differences in movement coordination patterns based on whether they are holding the conversation in person or over videoconference (e.g., Romero & Paxton, 2023; Zubek et al., 2022) or if a person violates a social norm (e.g., Miles, Griffiths, Richardson, & Macrae, 2010). In other words, interpersonal movement coordination appears to be a context-sensitive phenomenon – one that emerges differently in different conditions (e.g., Dale, Fusaroli, Duran, & Richardson, 2013; Paxton, 2026; Riley, Richardson, Shockley, & Ramenzoni, 2011).

**Table 1:** Questions to assess prior relationship before dyadic conversation. Note that option (b) for Question 1 was a compound option (i.e., “Maybe / Not sure / A little”), not three separate options

	Question text	Answer options	Branching
1	Did you know your partner prior to this study?	Choose one: (a) Yes; (b) Maybe / Not sure / A little; (c) No	All participants
2	How would you describe your knowledge of your partner?	Choose one: (a) Acquaintance (b) Classmate (c) Friend (d) Best friend (e) Romantic partner (f) Relative	Only if “yes” to Question 1
3	How long (in years) have you known your partner?		Only if “yes” to Question 1
4	How well do you know them?	On a scale from 0 to 100	Only if “yes” to Question 1
5	How close are you?	On a scale from 0 to 100	Only if “yes” to Question 1

Within the broad range of potential conditions that could impact interpersonal movement coordination, one relatively under-studied aspect of the context is the relationship between participants.^2^ Most prior work on interpersonal movement coordination has brought together strangers (i.e., people with no prior relationship), including pairs of true (naive) participants (e.g., Romero & Paxton, 2023), pairs with one true participant and one confederate (e.g., Miles et al., 2010), or pairs with one true participant and an experimenter (e.g., Hove & Risen, 2009). Although prior work has explored movement synchrony as an outcome or predictor among people with pre-existing relationships – like in romantic partners (e.g., Cohen, Abargil, Ahissar, & Atzil, 2024; Vargas-Quiros, Kapcak, Hung, & Cabrera-Quiros, 2021) or therapeutic relationships (e.g., Mende & Schmidt, 2021; Ramseyer & Tschacher, 2011) – very few prior studies have investigated the differences in interpersonal synchrony related to friendship.

Friendship is critically important for human wellbeing, but it is woefully understudied in psychological science (e.g., Dunbar, 2018). It is unsurprising, then, that friendship has been similarly understudied in the context of interpersonal synchrony. One recent study compared movement synchrony between friends and strangers, and although there was only a non-significant trend in the differences in the amount of synchrony, synchrony predicted later ratings of closeness *only* for strangers (Fujiwara, Kimura, & Daibo, 2020). Another study investigated the link between friendship and closeness during a support-seeking conversation and found an *inverse* relation between synchrony and feelings of support for people who self-identified only “friends” as compared with “close friends” (Lin et al., 2023). Given how little work has been done in this area, future study is needed to better understand the impact that prior relationship has on interpersonal synchrony. The present study is aimed at helping bridge this gap.

### Method

#### Participants

The example study consisted of 206 undergraduate students recruited through word-of-mouth, a digital bulletin board, and a participant pool system at the college. Participants received either class credit or $10 for their participation in the study. Participants’ ages ranged from 18 to 24 (*M* = 20.07 years, *SD* = 1.39). Participants were randomly paired up in the laboratory for a total of 103 dyads. Of these, 44 dyads reported having a previous relationship with each other prior to the study, and 59 dyads reported not knowing each other before arriving at the lab.

#### Materials

##### Questionnaire: Assessing pre-existing participant relationship

To assess participants’ previous relationship with each other before their participation in the study, each participant was separately asked to complete the questions provided in Table 1. Note that questions about the previous relationship (e.g., quality, duration; Questions 2–5) were only asked to participants if they indicated that they did know their partner before the study (Question 1).

##### Operationalization: Pre-existing relationships conditions

Using their responses to Table 1, we counted dyads whose participants answered “yes” to Question 1 as having pre-existing relationships with one another, which we will refer to as the “friends” condition. No participants indicated being relatives or romantic partners. Participants who answered anything else to Question 1 were grouped together in the “strangers” condition. No dyads reported mixed status between “friends” and “strangers” conditions. (All dyads counted as “friends” included participants who mutually indicated friendship; that is, all dyads in the “friends” condition *both* answered “yes” to Question 1 and *both* selected either ”Friend” or ”Best Friend” in Question 2.)

##### Conversation prompt

For the conversation task, each dyad was presented with the following prompt: *“What is one movie or book that you think everyone should experience at some point in their lifetime and why?”*. Participants were instructed to have a conversation about it for about six to eight minutes. Researchers informed participants when the allotted time had passed, while trying to find a natural stopping point in the conversation.

#### Procedure

Participants arrived to the lab and went through the informed consent process. Together, both participants in the dyad were given a brief introduction of the study session together in a hallway. Following this brief introduction to the study and their partner, they were led to separate rooms and asked to complete the informed consent forms. As part of this process, they also completed questions regarding how the videos collected in this project could be used in the future. Then, each participant completed the *Previous relationship questions* (see Table 1) on the survey platform Qualtrics individually in separate rooms.

**Fig. 3:**
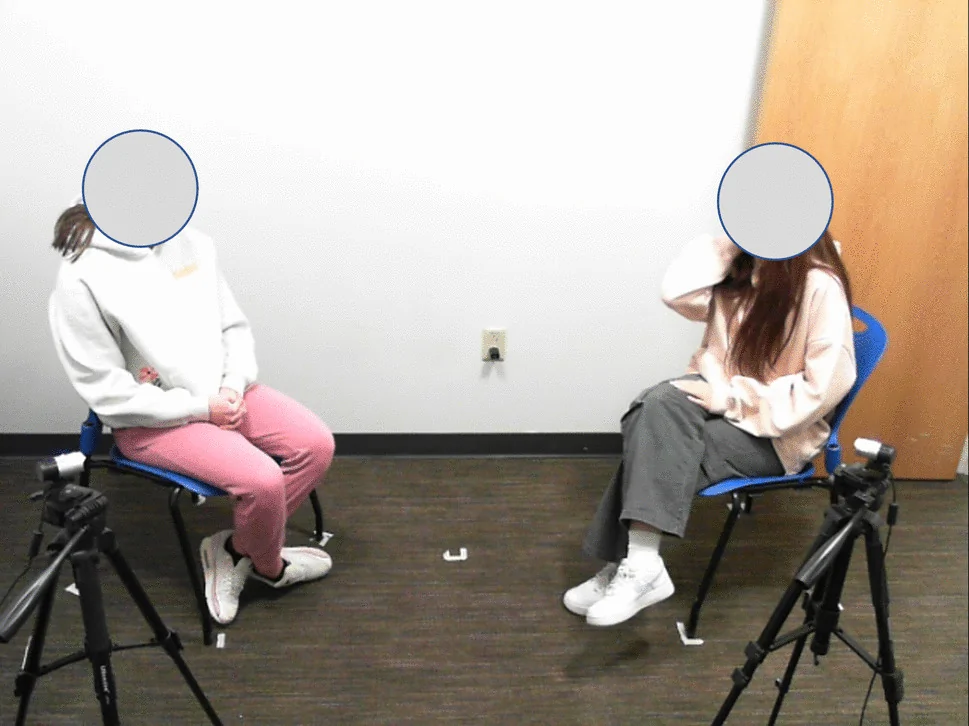
The view from one of the face-on web cameras used in the study. Photo provided with permission from the participants

Once all forms were completed, participants were brought together in another small, private room in the laboratory to have the *Conversation* described above. They were invited to sit on stationary chairs facing one another at a slight angle (see Fig. 3). Chairs were roughly 4 ft apart from one another, measured from the front of each seat. Participants were not told that they could move the chairs before sitting. If participants were strangers before the experiment, researchers asked participants to briefly introduce themselves to one another before beginning the conversation; otherwise, the conversation task began immediately.

During the interaction, participants were filmed using three web cameras (Microsoft LifeCam Studio 1080p HD Webcam). One web camera was placed above at an angle so it could capture both participants’ behavior simultaneously. The second and third webcams were placed so each could capture one participant face-on. The program MultiRec (https://multirec.app/) was used to capture all three cameras simultaneously on one computer, ensuring time-locking between all recordings.

Following their conversation, participants returned to their original rooms and completed a *Post conversation feelings* questionnaire individually. After the conversation task and related questionnaires, participants then completed other tasks unrelated to this publication. Finally, participants provided demographic information and were debriefed.

### Data processing and analysis

The Toolbox is equipped to extract movement from raw video data, but once the data has been extracted, it is up to the researchers how to analyze it. Below, we share our process for analyzing this data set, provided as a detailed example of *one* way of using the Toolbox and its output as a specific scientific project. We have decided to quantify interpersonal movement coordination using *cross recurrence quantification analysis* between participants within each dyad, followed by Mann–Whitney *U* tests to quantify differences between groups. Those interested in performing similar analyses can find the de-identified data and all analysis code in our OSF repository here: https://osf.io/9v86x/overview

#### Movement extraction

We used the “*Extract Pose Features*” function of the Toolbox to generate the movement time series for each participant from the two face-on video cameras. As described in the overview of the Toolbox (see Section “An open-source Toolbox for quantifying interaction,” above), this returned separate comma-separated files for each video.

**Fig. 4:**
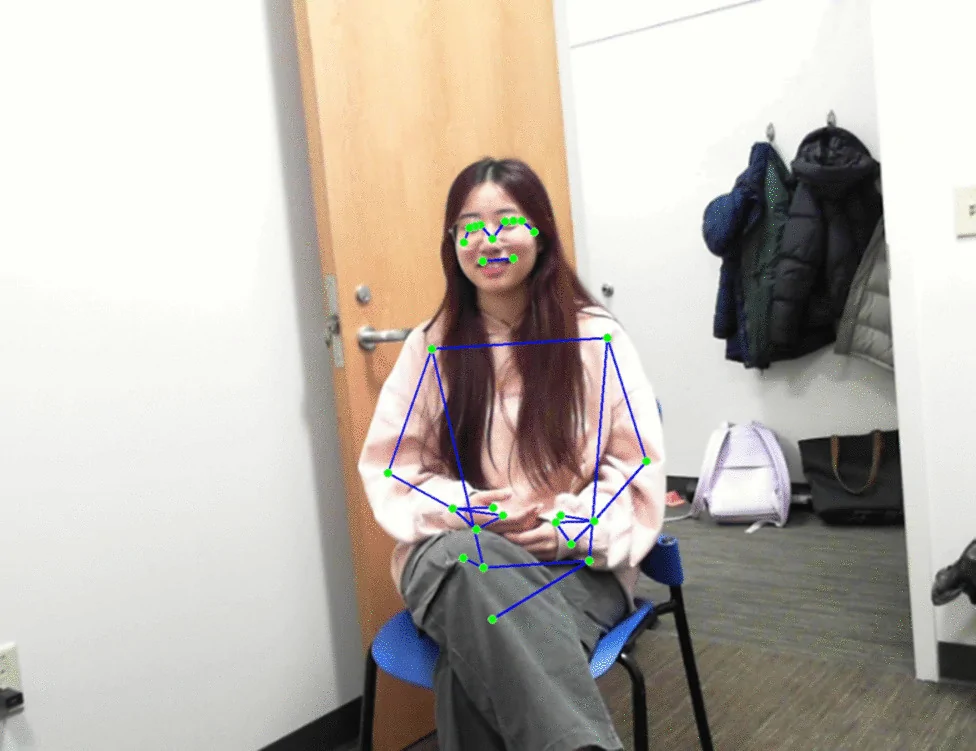
Example of one frame of one video with the embedded pose estimation. Photo provided with permission of the participant

For this study, we used the *Nose* and *Shoulder* landmark points in three-dimensional space. Using the *Nose* landmark, we calculated each person’s Euclidean trajectory. Using the *Left Shoulder* and *Right Shoulder* landmarks, we created a new measure to track each person’s Euclidean neck trajectory by calculating the midpoint between the left and right shoulders. These markers were chosen based on previous publications in the field (e.g., Zubek et al., 2022). For this example study, we used the separate straight-on recordings of each participant to minimize occlusion issues. For a visualization of how this worked, find an example of one frame with the embedded pose in Fig. 4

#### Recurrence quantification analysis

In order to measure the level and patterns of coordination between the participants during their conversations, we used the movement timeseries (extracted through the Toolbox) as input for *cross-recurrence quantification analysis* (*CRQA*; Coco & Dale, 2014; Webber & Zbilut, 2005). CRQA is a nonlinear time series analysis method that is able to quantify not only the level of connectedness or coordination between two time series, but also the patterning, stability, and complexity of that interconnectedness. This is a method that has been used in psychology to quantify interpersonal coordination among interacting individuals and how that coordination might be affected by different social and contextual variables (e.g., Romero & Paxton, 2023; Paxton & Dale, 2017; Zubek et al., 2022; Shockley, Santana, & Fowler, 2003).

CRQA measures the similarity between two time series by identifying when specific behavioral states reoccur over time. Additionally, the method quantifies the dynamic patterns of these recurrences. While the method extracts a number of metrics, we will focus on three of them: *recurrence rate* (*RR*), *maximum line length* (*MaxL*), and *entropy* (*ENTR*). For the purpose of our research questions, RR gives us a general measure of level of interpersonal coordination between the two participants’ nose and neck movement over the course of the entire conversation. Then, MaxL gives us a measure of the stability of this interpersonal bodily coordination, such that with longer MaxLs we find more stable patterns of coordination. Finally, ENTR is able to measure the level of complexity of the observed interpersonal coordination, with higher numbers indicating more complex patterns of coordination between participants. (For more on the interpretation of these and other metrics, see Coco & Dale, 2014; Pellecchia, Shockley, & Turvey, 2005; Webber & Zbilut, 2005)

For each pair, we performed conducted CRQA separately for each pair’s nose time series and their neck time series. As part of running CRQA analyses with continuous data (like frame-to-frame movement in Euclidean space), we chose to use different *delay*, *embedding dimension*, and *radius* parameters during phase-space reconstruction for each landmark. In keeping with typical procedures in the field (e.g., Coco & Dale, 2014; Romero & Paxton, 2023), we first calculated optimal delays for each pair of participants using the “average mutual information” (AMI) method and then the embedding dimensions using the “false nearest neighbors” (FNN) method. Additionally, each participant’s changes in movements were *z*-scaled within their own timeseries, and we used the *mean* method of rescaling in preparation to construct the recurrence plots (Webber & Zbilut, 2005).

Then, we calculated the mean of each of these parameters for the whole set of pairs in each landmark, such that we could use global parameters in order to draw conclusions about the differences between groups. Finally, the radius was calculated such that most of the pairs had an *RR* around 3%, as is customary in the field (Webber & Zbilut, 2005). For the nose landmark, the data presented below were obtained with a delay of 8, an embedding dimension of 5, and a radius of 0.3; for the neck landmark, we used a delay of 8, an embedding dimension of 5, and a radius of 0.2. To ensure the results were not artifacts of parameter selection during phase-space reconstruction, we conducted the same analyses using two other sets of parameters for each landmark, and we obtained consistent results.

#### Statistical analysis

The CRQA metrics in our dataset were not normally distributed. As a result, we used a series of Mann–Whitney *U* tests to uncover the differences between interpersonal movement coordination for friends versus strangers. These analyses were conducted using JASP (Jasp Team, 2025).

A common method of testing statistical significance for recurrence-based measures includes the construction of an appropriate *surrogate dataset*, which serves as way of testing how much recurrence might be expected to occur by chance. Typically, researchers do this by taking the original time series (often called the “real” time series) and manipulating it in one of several ways. A *sample-wise shuffled baseline* breaks the temporal dependence of the real time series by re-ordering the observations within each dyad to create surrogate time series; it can be especially helpful when accounting for dyads’ idiosyncratic baseline levels of a given behavior within the dataset. A *surrogate pairs baseline* retains the temporal ordering of each participant’s behavior but breaks the interpersonal dependence by creating “false pairs” of two participants who never actually interacted; this is helpful when accounting for the structure of the task in driving recurrence. Other baseline measures also exist to account for other statistical properties of the dataset and are explored elsewhere (e.g., Paxton & Dale, 2017). However, in cases in which the overall *amount of recurrence* is less of a focus than the *differences in recurrence between conditions*, the comparison between conditions often suffices (as we do here).

### Results

#### Interpersonal coordination of the nose

**Fig. 5:**
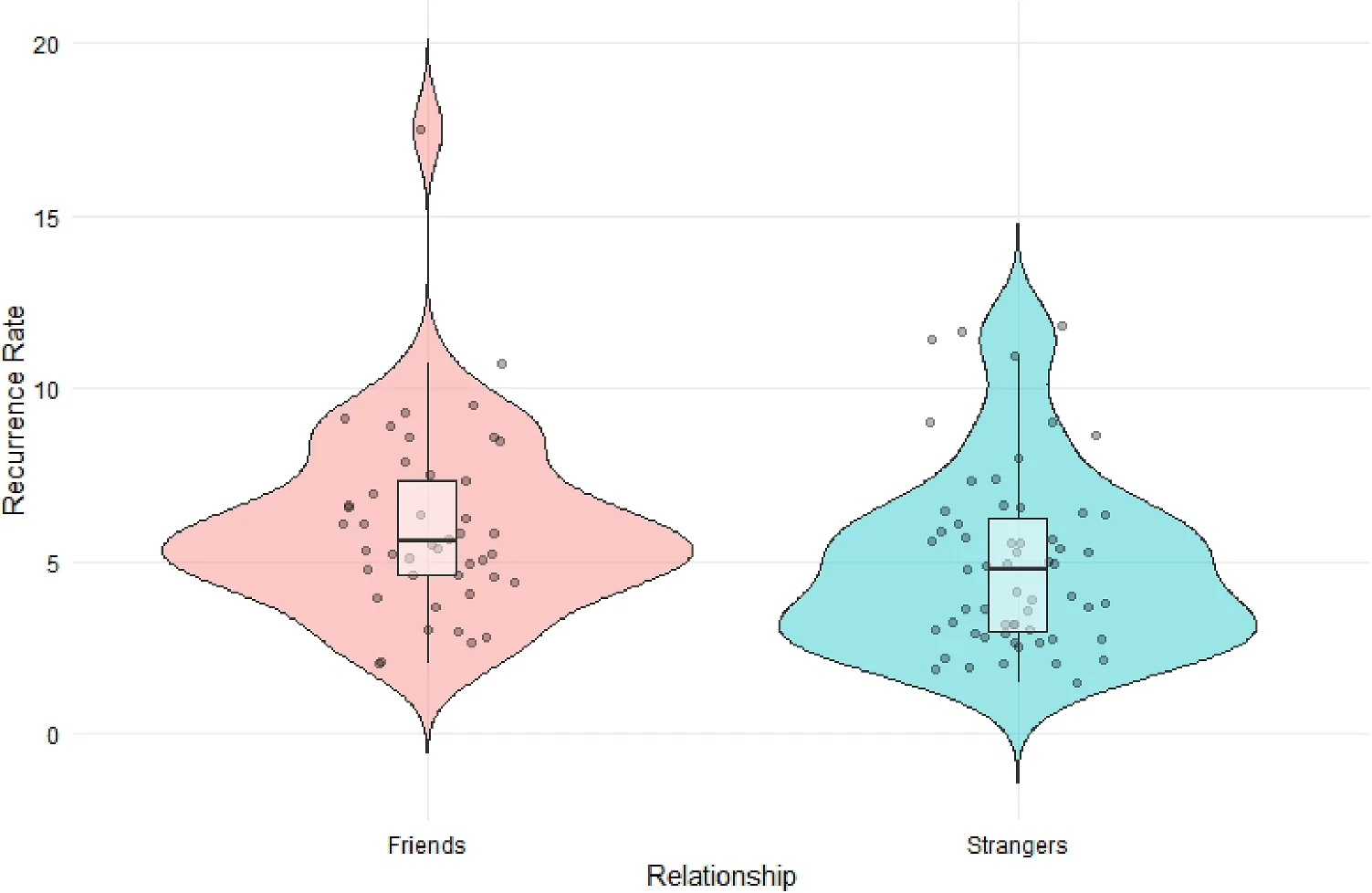
Violin plots of mean recurrence rate (RR) of the nose by previous relationship of the dyad

Friends had significantly higher nose RR (*M* = 6.09, *SD* = 2.75) than strangers (*M* = 4.97, *SD* = 2.59), *U* = 1649, *p* = .02 (see Fig. 5). This indicates that friends had higher overall head movement coordination during the conversation.

**Fig. 6:**
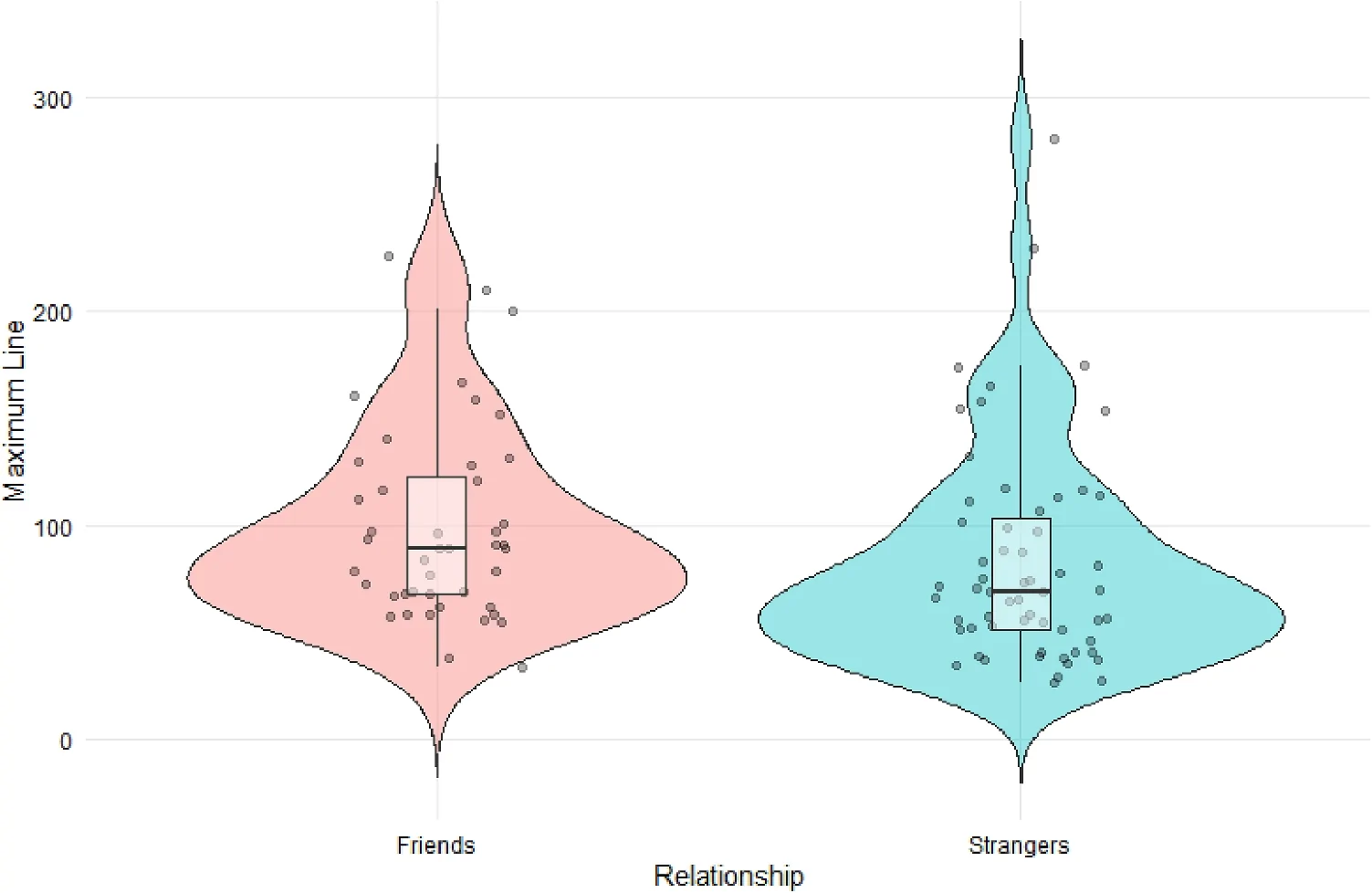
Violin plots of mean maximum line (MaxL) by previous relationship of the dyad

Friends also significantly higher nose MaxL (*M* = 98.91, *SD* = 45.38) than strangers (*M* = 82.81, *SD* = 50.98), *U* = 1664.50, *p* = .01 (see Fig. 6). This suggests that the friends’ head movement coordination was more stable, compared with strangers’.

**Fig. 7:**
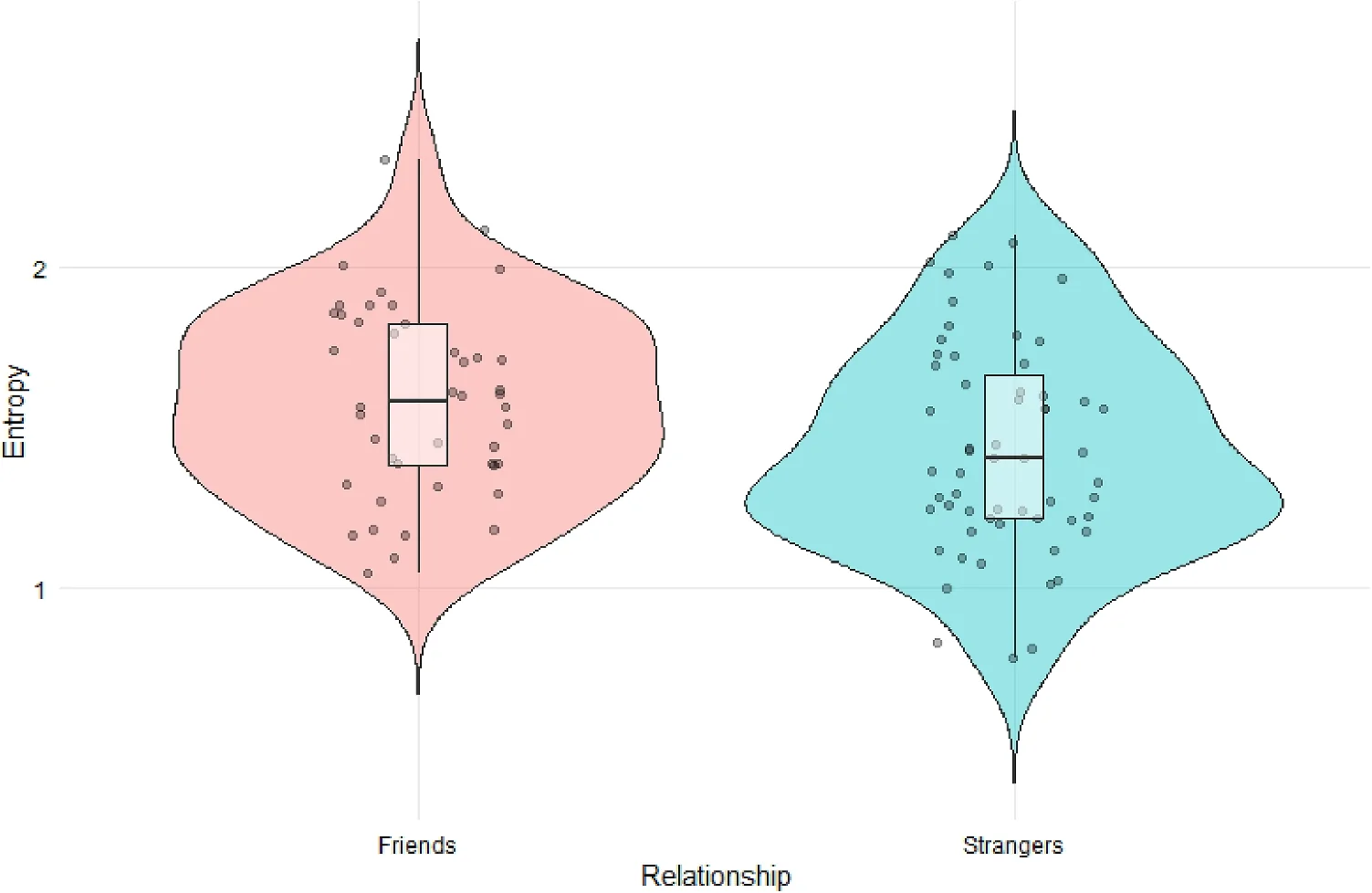
Violin plot of mean entropy (ENTR) of the nose by previous relationship of the dyad

Finally, friends also had significantly higher nose ENTR (*M* = 1.59, *SD* = 0.30) than strangers (*M* = 1.43, *SD* = 0.33), *U* = 1656, *p* = .02 (see Fig. 7). This means that friends’ head movement coordination had less predictable or more complex patterns than that of strangers.

#### Interpersonal coordination of the neck

**Fig. 8:**
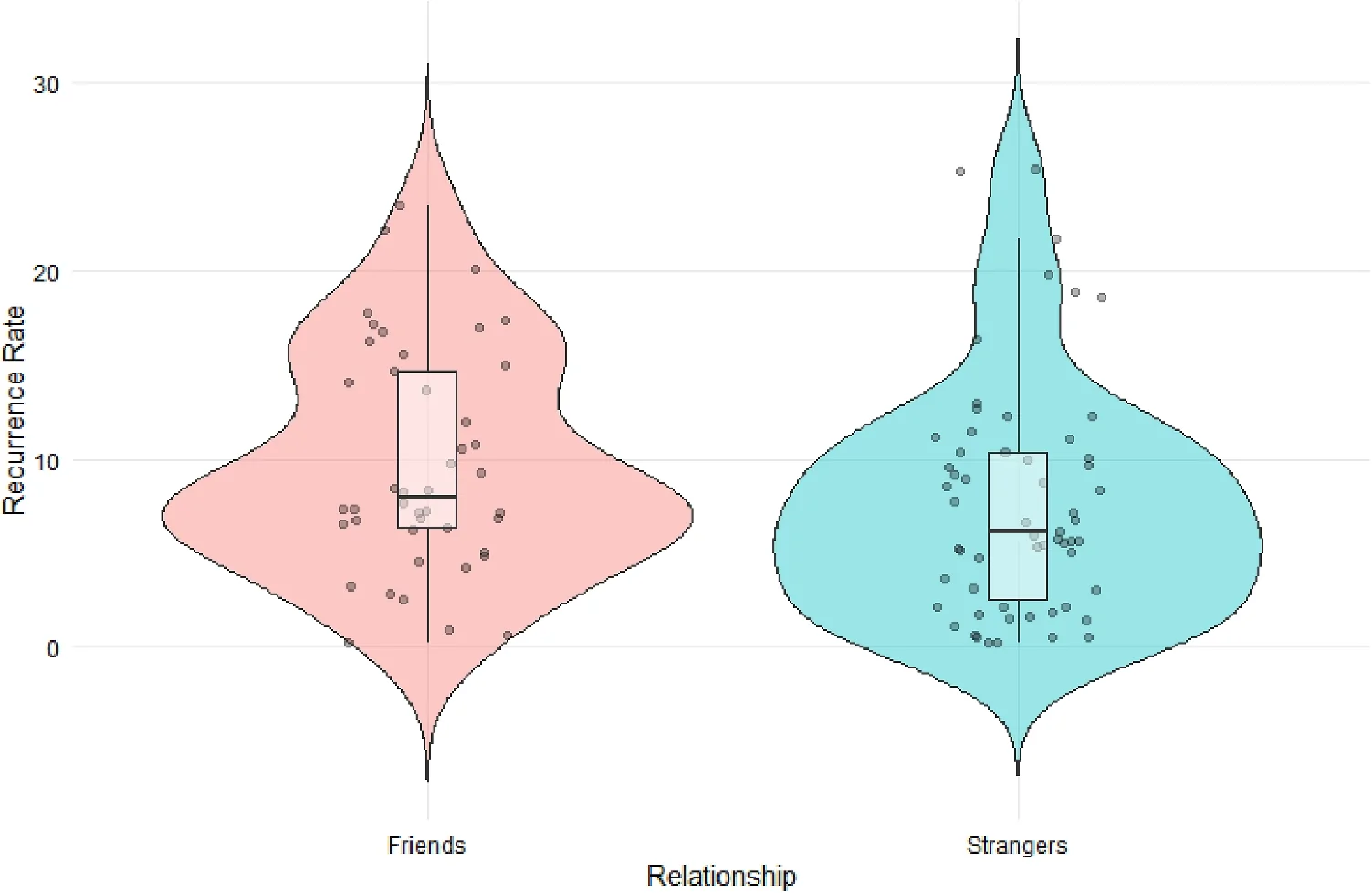
Violin plot of mean recurrence rate (RR) of neck movement by previous relationship of dyad

**Fig. 9:**
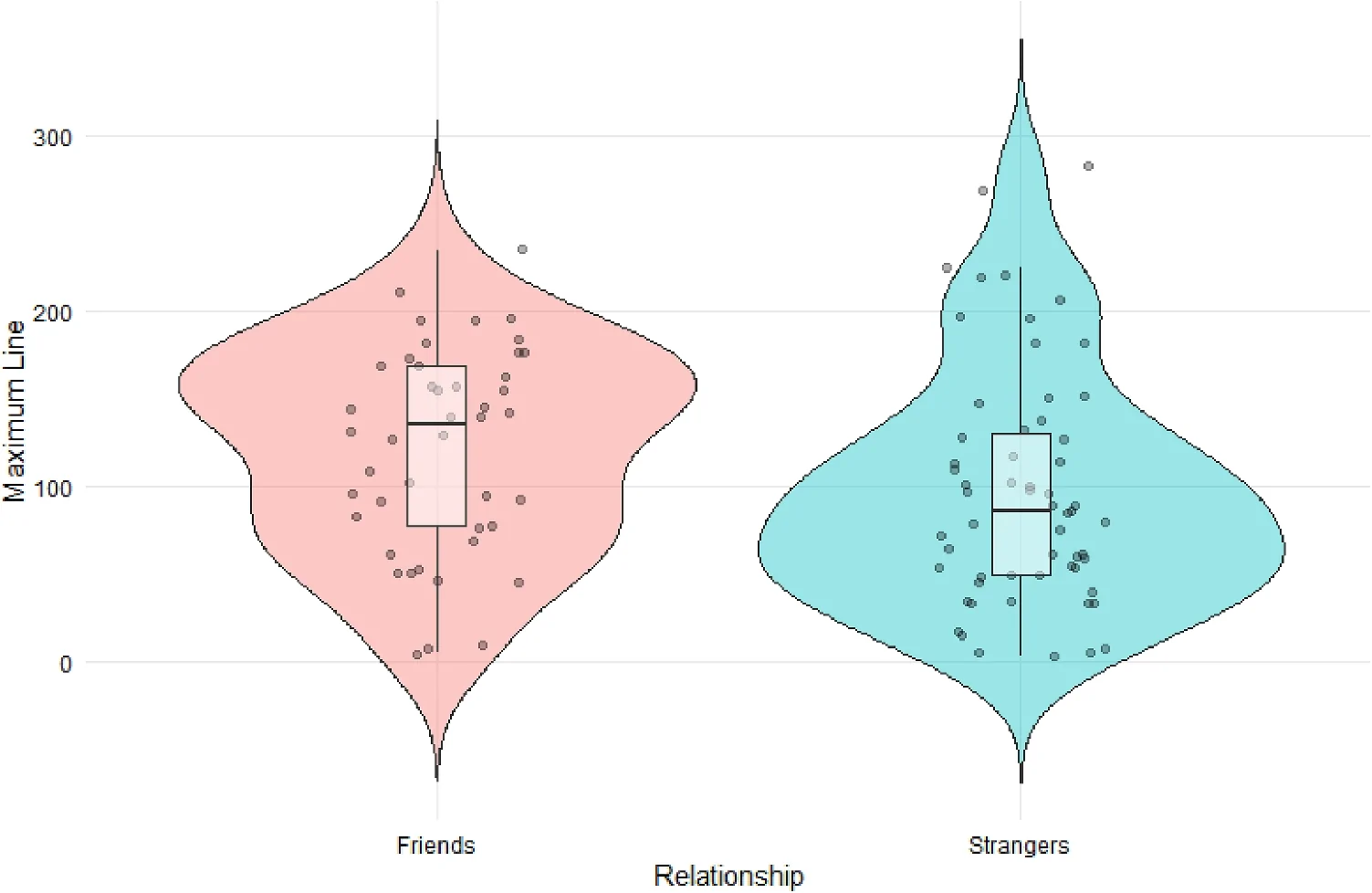
Violin plot of mean maximum line (MaxL) of neck movement by previous relationship of dyad

**Fig. 10:**
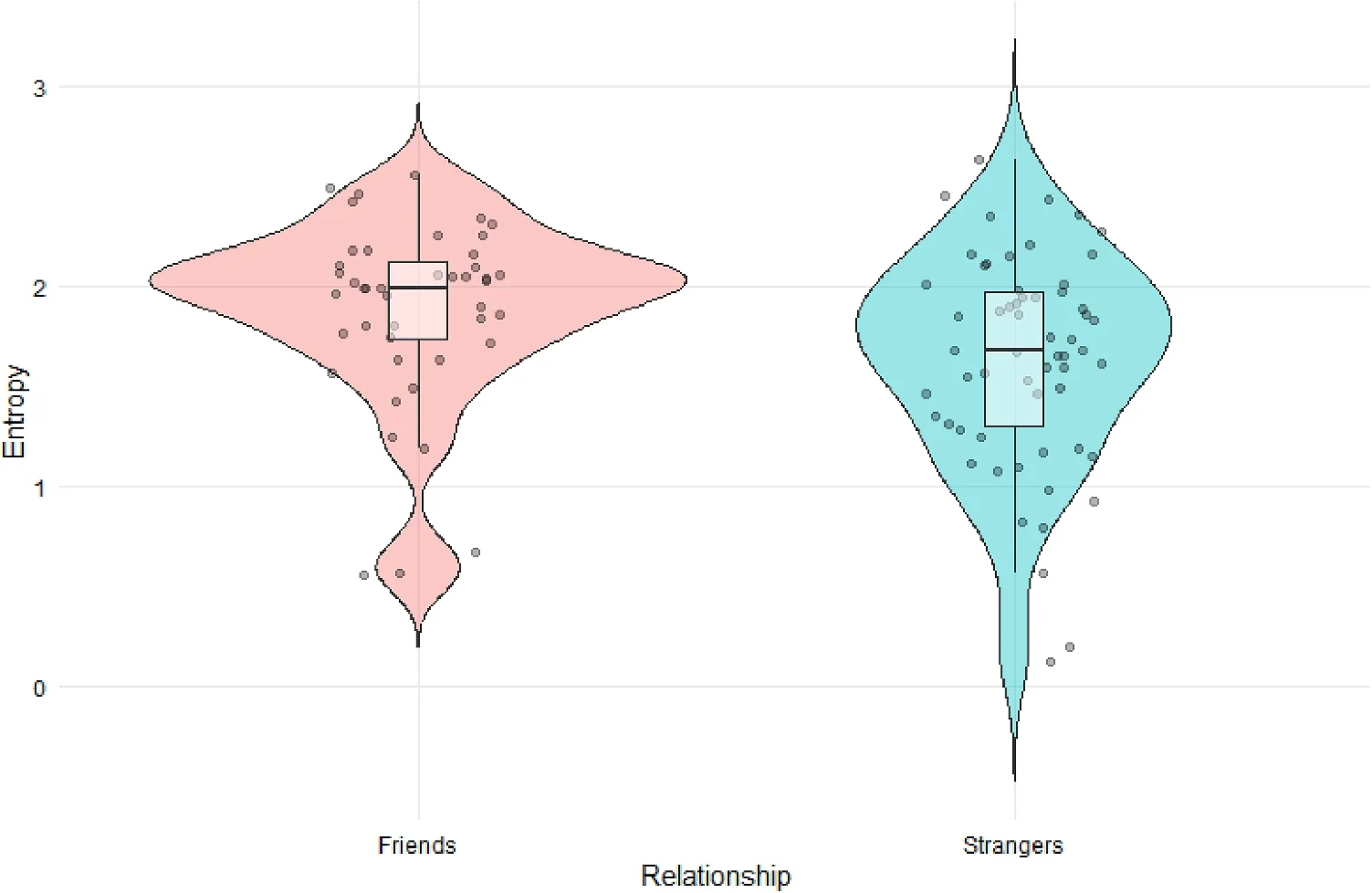
Violin plot of mean entropy (ENTR) of the neck by previous relationship of the dyad

Results of neck movements showed the same patterns as for head movements. Friends had significantly higher neck RR (*M* = 9.80, *SD* = 5.91) than strangers (*M* = 7.73, *SD* = 6.18), *U* = 1587, *p* = .05 (see Fig. 8). Friends’ neck MaxL (*M* = 121.91, *SD* = 58.61) was also significantly higher than strangers’ (*M* = 97.92, *SD* = 67.58), *U* = 1628, *p* = .03 (see Fig. 9). Friends had higher neck ENTR (*M* = 1.59, *SD* = 0.30), compared with than strangers (*M* = 1.43, *SD* = 0.33), *U* = 1656, *p* = .02 (see Fig. 10). Taken together, friends’ upper torso movements were generally more coordinated, more stably coordinated, *and* more complexly coordinated during conversation, compared to strangers’.

### Interim discussion

As part of demonstrating the utility and ease-of-use of the Toolbox, this example study set out to better understand the impact that prior relationship has on interpersonal coordination by comparing head and neck movements of friends versus strangers. Although we here report on our own analyses of the dataset, a portion of this dataset and tools were used by undergraduate students as part of their coursework for a one-semester class (led by the current paper coauthor V.R.), and they had no prior specialized training in these methods.

Interestingly, we found significant differences between the level of coordination, the stability of the coordination, and the complexity of that coordination based on previous relationship of the participants. Compared with strangers, friends’ head and torso movements were coordinated *more overall* and *more stably* – even though those patterns of coordination were simultaneously *more complex*. These results highlight the importance of asking participants about their previous relationship in future studies, as it seems that being friends could lead to significantly different coordination patterns than strangers (Dunbar, 2018).^3^

## Recommendations and practical considerations

We close by providing some suggestions and practical considerations for using the MultiSOCIAL Toolbox. We hope they will be helpful to people at all stages of the data collection pipeline – whether they are designing a study or hoping to use the Toolbox to analyze existing data. While some of these are limitations based on the Toolbox at the time of publication, we hope that future iterations of the Toolbox may help improve its capabilities as we develop more functionalities, flexibility, and versatility.

Critically, we want to first reiterate that the Toolbox is a means of *data extraction*: Researchers must bring a discerning eye and their knowledge of their phenomenon of interest when creating a study for use with the Toolbox *and* when using the Toolbox’s output for any kind of analysis. Above, we presented just one example use of the Toolbox, and we hope that others will use the Toolbox to support other pipelines. However, *all* methods – including ours – have strengths and limitations, and we urge all users to look carefully at their data along the way and adapt as necessary.

1. *Complexities and limitations of automated methods.* The MultiSOCIAL Toolbox is based on automated tool-based feature extraction from multimodal behavior and interaction data. These tools were often developed using data collected in a certain condition both behaviorally and environmentally (e.g., single person, non-overlapping speech, front-view recording). As a result, these tools are less reliable when these conditions do not hold, and we recommend researchers being mindful of these limitations. Knowing these limitations is important for anyone who wishes to use the Toolbox: Those who are collecting new data can address them in their setup, and those who are (re)analyzing existing data can proceed carefully with data extraction and preparation accordingly. In all cases, to improve data fidelity and study robustness, researchers should *always* critically examine the resulting data and, where necessary, manually correct the automatic methods’ results.
2. *Experimental setup for pose estimation.* All pose estimation – including the Toolbox – is sensitive to occlusion of participants’ body parts (e.g., if people appear to overlap, if part of a person’s body is blocked by an object), camera angle, and lighting conditions. All of these can lower pose estimation accuracy for MediaPipe (Fan & Chowdhury, 2025). For example, a camera placed at a central location capturing all participants can miss subtle body movements occurring at oblique angles or outside of the camera view. To improve these limitations, we recommend using a standardized camera setup so that participants’ full bodies are visible without frequent occlusion in a well-lit condition to minimize the pose estimation error. While this setup is not immune to issues (since participants may still occlude some landmarks while moving and occupy different portions of the frame), it goes a long way to minimizing issues when setting up the data collection pipeline. However, researchers still should be vigilant with the extracted data and complete the necessary pre-processing steps for their chosen analysis methods.
3. *Experimental setup for audio features and transcript creation.* Speech processing tools – including those used in the Toolbox – have designed for machine learning and phonetic research, which means that behavioral features extracted by different automated tools from the same audio file can differ among themselves (Chowdhury & Romero, 2025). Additionally, overlapping speech can cause problems for data extraction if audio from multiple participants are recorded to a single channel, because it combines signals from all participants as a mono-channel audio that can be difficult for automated tools to separate. Therefore, we recommend capturing participants’ audio using separate microphones; clean audio is especially important when overlapping speech is expected, as often happens in conversation. When possible, recording each speaker on a separate channel (rather than mixing all voices together) can make both transcription and diarization more accurate. Any improvement in the automated method will accordingly reduce post-processing steps and human verification time.
4. *Experimental setup for multi-person data collection.* The Toolbox can currently handle single-participant and multi-person recordings. Multi-person video setup can introduce additional complexity for individual person tracking and synchronization, which we attempted to address by combining multiple tools such as object detection, tracking, and pose estimation. However, as with any research method, the extracted data may still have errors. For example, when using the Toolbox for pose estimation for a different study (which included dyads doing yoga together), the algorithm would sometimes detect more than two people in the frame or would suddenly “swap” skeletons between people (such that formerly Participant 0 became Participant 1 and vice-versa). Therefore, for multi-person pose estimation, we recommend recording in well-lit spaces with well-separated seating arrangements; not only will this improve multi-person detection, but it can also open opportunities for further “data rescue” if systemic problems emerge (e.g., by cropping videos so that only a single participant is visible at a time and running single-participant pose estimation over each). Similarly, for audio recordings, we recommend including using a separate microphone for each person, followed by manual correction of the automated transcription output. When recording across multiple devices (for video and/or audio), be sure to synchronize recording devices either with a technical solution (e.g., LabStreamingLayer; Kothe et al., 2025) or a manual one (e.g., a clapperboard).
5. *Filetype requirements and recommendations.* The Toolbox accepts the video formats .mp4, .avi, .mov, .mkv, and .m4v, as well as common audio formats including .wav, .wave, .aiff, .aif, .aifc, .flac, .caf, .au, and .snd. Its built-in conversion step uses FFmpeg (Tomar, 2006) to convert any supported video file to .wav audio; it is therefore not limited to .mp4-to-.wav conversion. Recordings in unsupported formats should be batch-converted with FFmpeg or another suitable tool before modality-specific processing. We recommend selecting consistent recording formats during data collection to reduce avoidable file-management and processing errors in large batches.
6. *Community contribution and open-source development.* We envision this Toolbox growing in both functionalities and user base over the years as the behavioral research community finds new ways and new tools useful for this line of research. The codebase for the Toolbox is openly available on GitHub^4^, and we welcome contributions from the community to extend the Toolbox. (For example, someone might help by adding a custom feature extraction recipe for a specific context or improving user interface components to support cross-tool comparison.) If a user adapts the main codebase for a specific research need, we encourage researchers to contribute back to MultiSOCIAL via a pull request to the official code repository. Even small fixes (e.g., adding a new converter or clarifying documentation) can benefit others. Such collaborative development would accelerate refinement and adoption across behavioral research contexts. This will also enable inspection and validation of automated tools’ output in diverse conditions and populations, which can reveal new insights into their reliability and validity across contexts.

## Data Availability

The de-identified data and materials for all experiments are available at https://osf.io/9v86x/overview.

## Footnotes

1 OpenSMILE documentation provides more details about the applicability of each of its three feature sets at https://audeering.github.io/opensmile-python/

2 Other work has also studied the effects of prior relationships on other kinds of synchrony, like physiological synchrony (e.g., Bizzego et al., 2019; Ma & Liu, 2024)]. Because different kinds of synchrony are hypothesized to serve qualitatively different functions (e.g., Paxton, 2026), we restrict our review strictly to movement synchrony.

3 While we collected some data about the subjective feelings of participants following the conversation, this is beyond the scope of the current article, so we cannot at this time add to the findings by Fujiwara et al. (2020) and Lin et al. (2023).

4 https://github.com/Tahiya31/MultiSOCIAL_Toolbox/

## Code Availability

The de-identified data and materials for all experiments are available at https://osf.io/9v86x/overview.

## Declarations

### Open Practices Statement

The de-identified data and materials for all experiments are available at https://osf.io/9v86x/overview. The experiment was not preregistered.
